# Development of a thyroid cancer prognostic model based on the mitophagy-associated differentially expressed genes

**DOI:** 10.1007/s12672-023-00772-6

**Published:** 2023-09-14

**Authors:** Wencong Sun, Xinhui Wang, Guoqing Li, Chao Ding, Yichen Wang, Zijie Su, Meifang Xue

**Affiliations:** 1 Department of Thyroid Surgery, Henan Provincial People’s Hospital, Zhengzhou University People’s Hospital, Zhengzhou, Henan China; 2 Department of Geriatric, Henan Provincial People’s Hospital, Zhengzhou University People’s Hospital, Zhengzhou, Henan China; 3 Health Management Section, Zhumadian Central Hospital, Zhumadian, Henan China

**Keywords:** Thyroid cancer, Prognosis, Mitophagy, TCGA

## Abstract

**Background:**

The prevalence of thyroid cancer (ThyC), a frequent malignant tumor of the endocrine system, has been rapidly increasing over time. The mitophagy pathway is reported to play a critical role in ThyC onset and progression in many studies. This research aims to create a mitophagy-related survival prediction model for ThyC patients.

**Methods:**

Genes connected to mitophagy were found in the GeneCards database. The Cancer Genome Atlas (TCGA) and Gene Expression Omnibus (GEO) databases provided information on the expression patterns of ThyC-related genes. To identify differentially expressed genes (DEGs), R software was employed. The prognostic significance of each DEG was assessed using the prognostic K-M curve. The prognostic model was built using LASSO, ROC, univariate, and multivariate Cox regression analyses. Finally, a nomogram model was developed to predict the survival outcome of ThyC patients in the clinical setting.

**Results:**

Through differential analysis, functional enrichment analysis, and protein–protein interaction (PPI) network analysis, we screened 10 key genes related to mitophagy in ThyC. The risk model was eventually developed using LASSO and Cox regression analyses based on the six DEGs related to mitophagy. An altered expression level of a mitophagy-related prognostic gene, *GGCT*, was found to be the most significant one, according to the KM survival curve analysis. An immunohistochemical (IHC) investigation revealed that ThyC tissues expressed higher levels of GGCT than normal thyroid tissues. The ROC curve verified the satisfactory performance of the model in survival prediction. Multivariate Cox regression analysis showed that the pathological grade, residual tumor volume, and initial tumor lesion type were significantly linked to the prognosis. Finally, we created a nomogram to predict the overall survival rate of ThyC patients at 3-, 5-, and 7- year time points.

**Conclusion:**

The nomogram risk prediction model was developed to precisely predict the survival rate of ThyC patients. The model was validated based on the most significant DEG GGCT gene expression in ThyC. This model may serve as a guide for the creation of precise treatment plans for ThyC patients.

**Supplementary Information:**

The online version contains supplementary material available at 10.1007/s12672-023-00772-6.

## Introduction

Thyroid carcinoma (ThyC) is one of the most frequently diagnosed endocrine malignancies in adults, accounting for approximately 96.0% of all newly diagnosed endocrine cancers and involving females in 77% of cases [1]. Globally, there were around 580,000 newly diagnosed ThyC cases in 2020, thereby ranking this carcinoma 11^th^ among all types of cancers in humans [2]. ThyC can be histopathologically classified into four major subgroups based on the cancer-origin cell type, namely papillary (PTC), follicular (FTC), medullary (MTC), and anaplastic thyroid cancer (ATC). Of these, PTC is the most frequent kind and exhibits a favorable prognosis [3–5]. Although surgery is currently the major form of treatment for ThyC patients, however, there are still cases (< 10%) of ThyC patients present rapid progression of cancer symptoms and poor outcomes in surgical removal as well as the worst prognosis [6]. Multiple risk factors may also affect the prognostic outcomes in these patients, for example, the pathological stage, the extent of invasion, lymph node metastasis, and residual tumor volume. Since the pathogenesis of ThyC is complex, it is essential to establish a standardized prognostic evaluation model [7].

Thyroglobulin (Tg) and calcitonin are commonly used as tumor biomarkers for several years regarding the postoperative follow-up evaluation of ThyC, according to the guidelines of the American Thyroid Association (ATA) [8]. However, Tg shows a low specificity and is only indicative of cancer progression in patients with progressively elevated Tg levels after the surgical resection. Calcitonin, as a specific indicator of MTC, has an important value for tumor screening and prognostic evaluations. Recently, a slew of molecular markers, such as BRAF [9], RAS [10], RET-PTC [11], TERT [12], and other gene mutations and/or rearrangement, have been proposed to facilitate the diagnosis and prognosis of ThyC patients. Although these genes have not yet entered the clinical first line to guide prognosis, the screening of mutations in multiple genes may cumulatively serve as an important indicator toward prognostic evaluations.

An evolutionarily conserved mechanism, called autophagy, allows cells to discard/recycle expired or damaged components mainly through the lysosomal degradation pathway [13]. Under normal physiological conditions, a mitochondria-specific autophagic pathway, mitophagy, is triggered in response to multiple stressors such as food scarcity, hypoxia, DNA damage, inflammation, and mitochondrial membrane depolarization [14, 15]. PARK2, FANCC, BNIP3, and BNIP3L are among those mitophagy regulators that are aberrantly expressed during malignancies [16]. Recent studies have demonstrated that mitophagy pathway dysregulation can modulate the prognosis of ThyC [17–20].

By exploiting ThyC-associated mRNA expression profiles and the TCGA and GEO-derived clinical data, we identified a set of differentially expressed genes (DEGs) involved in mitophagy and subsequently constructed a risk prediction model for ThyC patients. In parallel, we validated the prognostic model for its precision, reliability, and reproducibility for risk prediction.

## Materials and methods

### Data acquisition

The TCGA data portal, containing 510 ThyC and 58 neighboring non-tumor tissue transcriptomics profiles, was used to retrieve RNA-seq data for ThyC [21]. Simultaneously, the UCSC Xena database was used to retrieve any relevant clinical data (http://genome.ucsc.edu) [22]. The ThyC microarray data (GSE3678) was also downloaded from the GEO database [23]. The data platform for this dataset was the GPL570 Affymetrix Human Genome U133 Plus 2.0 Array. We included array profiles of seven specimens for each of the ThyC and the matched control groups from this database. In total,2,414 mitophagy-related genes expression profiles were retrieved from the GeneCards database(https://www.genecards.org/) [24]. Table S1 describes expressions of analyzed genes.

### Identification of ThyC-associated DEGs

To pinpoint the underlying mechanism, associated biological traits, and DEG-related pathways in ThyC, we first used the limma [25] package to normalize TCGA-ThyC and GSE3678 datasets. The TCGA-ThyC dataset's count data were then subjected to differential analysis using R-based DESeq2 [26], while the GSE3678 expression profile data were differentially analyzed using the R software limma. We obtained two ThyC data sets from different DEG groups, and genes with |log2FC|> 1 and adjusted P ≤ 0.05 were considered statistically significant.

To identify DEGs that were associated with mitophagy in ThyC, we first analyzed the intersection points of all DEGs in the TCGA-ThyC and GSE3678 datasets and plotted the Venn diagram to acquire common DEGs in these data sets. Then co-DEG and mitophagy-related genes were interfaced by plotting, a Venn diagram. The heatmap and volcano plot were created using the R tool ggplot2 and the findings of the differential analysis.

### Functional enrichment analysis

Studies on functional enrichment at a large scale, encompassing biological process (BP), molecular function (MF), and cellular components (CC), are frequently carried out using Gene Ontology [27] (GO). We used the R package clusterProfiler [28] to execute the GO annotation analysis on mitophagy-related DEGs. The entrance screening criteria were *P* and FDR values less than 0.05 for statistical.

### Gene set enrichment analysis (GSEA)

To assess the contribution of disease-relevant gene expressions to the phenotype, the gene distribution tendency of a pre-assorted gene set in the dataset was evaluated using the GSEA [29]. Based on the degree of phenotypic connections, genes from the TCGA-ThyC and GSE3678 datasets were first split into two groups to perform the enrichment analysis on all DEGs with the clusterProfiler program using the following settings seed = 2,020; computations = 1,000; the minimum amount of genes in apiece gene set = 10, the maximum amount of genes in apiece gene set = 500, and Benjamini–Hochberg P-value correction (BH). Both P and FDR values of less than or equal to 0.05 were considered statistically significant for the gene set h. all.v7.2.Symbols.gmt, obtained from the Molecular Signatures Database (MSigDB) [30].

### Construction of a protein–protein interaction network (PPI)

The PPI network was composed of individual proteins interacting with each other. A database to explore the connection between predicted and experimentally validated proteins is called the STRING database [31]. We constructed a PPI network with the STRING database for the selected mitophagy-associated DEGs, and the PPI network model was constructed using Cytoscape [32]. The maximal clique centrality (MCC) algorithm [33] has been widely utilized as a performance metric in bioinformatics. PPI networks with tightly connected and, tiny areas might include chemical compounds with specific biological activities. The PPI network scores of mitophagy-related DEGs that were linked to other PPI network nodes were mined using the MCC method. Finally, the top ten mitophagy-related DEGs were ranked according to the scores and were selected as the key genes (hub genes) for ThyC.

### Establishment of a mitophagy-related prognostic model

To develop a prognostic model of DEGs connected with mitophagy in ThyC, LASSO regression was performed using tenfold cross-validation with a *P*-value of 0.05. LASSO regression is often used to construct prognostic models. To minimize the overfitting effect and increase generalizability, the penalty term was introduced to the linear regression model. Following the visualization of the LASSO regression results, the risk factor map was used to further explain the grouping of each sample according to the survival outcome in the prognostic model as well as the molecular expression of prognostic DEGs related to mitophagy in each group.

The prognostic Kaplan–Meier (KM) curve analysis method, also known as survival analysis, is a way to analyze and infer the survival time of patients to explore the link between the survival time and outcome. It was proposed by Kaplan and Meier, so it is called the Kaplan–Meier method, often referred to as the KM method, as well. The KM survival curve method is usually used to calculate the survival probability—i.e., the likelihood that a patient who has survived for one period would also survive for the subsequent period—and multiplies these probabilities one at a time to get the survival rate for that period. The KM curve was plotted for mitophagy-associated DEGs in the LASSO model.

### The receiver operating characteristic (ROC) curve analysis

The ROC curve analysis [34] refers to a method for examining the coordinate schemas that can be employed to choose the right model, rule out a runner-up model, or determine the optimal threshold within the same model. The composition approach displays the relationship between sensitivity and specificity, and the ROC curve provides a full illustration of continuously varying representations of both sensitivity and specificity. The closer area under curve (AUC) is to 1, the stronger the diagnostic impact is. A range of AUC values from 0.5 to 0.7 indicates a low accuracy; values between 0.7 and 0.9 are considered for medium accuracy, and any values greater than 0.9 indicates the highest accuracy. We used the R software survivalROC package to draw the ROC curve and calculate the corresponding AUC to evaluate the contribution of mitophagy-associated DEGs to the survival of ThyC patients.

### Clinical correlation analysis of prognosis

To determine the predictive value of the identified mitophagy-related DEGs in ThyC, a univariate Cox regression analysis was performed using the gene expression profile and clinical features for each patient. Factors with a P-value of less than 0.01 were then included in the multivariate Cox regression model. Based on these findings, nomograms were created to predict the 3-, 5-, and 7-year survival rates of ThyC patients. The nomogram's accuracy and discrimination were assessed using the calibration curve. For the construction of the nomogram and calibration curve, the R package "rms" was applied. The decision curve analysis (DCA) was performed to assess the effect of the predictive nomogram model of ThyC using the R package ggDCA [35] to explore the possible survival outcomes in these patients.

### Gene set variation analysis (GSVA)

A nonparametric unsupervised analytical technique, called gene set variation analysis (GSVA) [36], is mostly applied to determine the gene set enrichment of target gene(s) in microarray and transcriptomics studies. For GSVA at the gene expression level, the gene set "h.all.v7.4.Symbols.gmt" was extracted from the MSigDB database to analyze the functional enrichment variations between the two tissues. This was done to identify if different tissues had different gene expression enrichment profiles within the same group.

### Immunohistochemical (IHC) analysis

Expressions of prognostic DEGs related to mitophagy in control versus ThyC tissues were analyzed by the IHC method using the Human Protein Atlas (HPA) [37] database (www.proteinatlas.org/) as the reference. The results of this analysis were included in the database.

### Statistical analysis

Employing R software, version 4.1.2, the whole data processing, and analysis for this report was completed. Continuous variables were expressed as means ± standard deviation (SD). The Wilcoxon rank-sum test was used to compare the two groups. To compare the two groups with at least three distinct sizes, the Kruskal–Wallis test was performed. The chi-squared (χ^2^), or Fisher's exact test was used to analyze the statistical significance between the two sets of categorical variables. If not otherwise specified, a P-value of less than 0.05 was considered statistically significant in all analyses. If not otherwise stated, the results were estimated as correlation coefficients between various groups using Spearman correlation analysis.

## Results

### Flow chart of this study

**Fig. 1:**
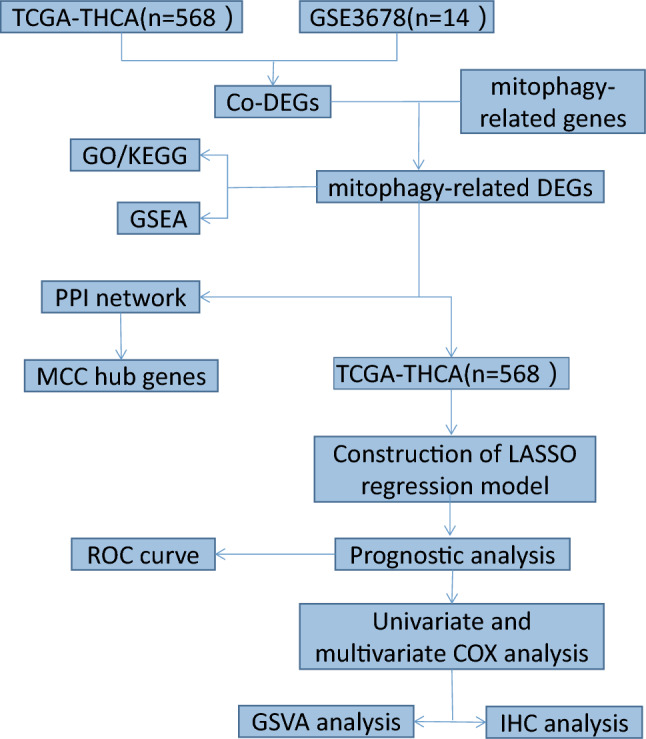
Flowchart of the identification of the mitophagy-related gene signatures in thyroid cancer (ThyC)

Figure 1 displays a thorough work flow diagram for this study. First, ThyC and GSE3678 datasets were respectively retrieved from the TCGA and GEO database's. Genes related to mitophagy were intersected with differentially co-expressed genes. For the discovered differentially co-expressed mitophagy-related genes, functional enrichment, and PPI network analyses were carried out. A prognostic model was then constructed based on clinically pertinent data using LASSO regression and KM curve analysis. To assess the clinical prognostic significance of ThyC-related DEGs, univariate and multivariate Cox regression models were employed. Finally, the difference in functional enrichment between the two groups was analyzed by GSVA, and the related DEGs were further analyzed by IHC in control and ThyC tissue samples.

### Analysis of mitophagy-associated DEGs in ThyC

**Fig. 2:**
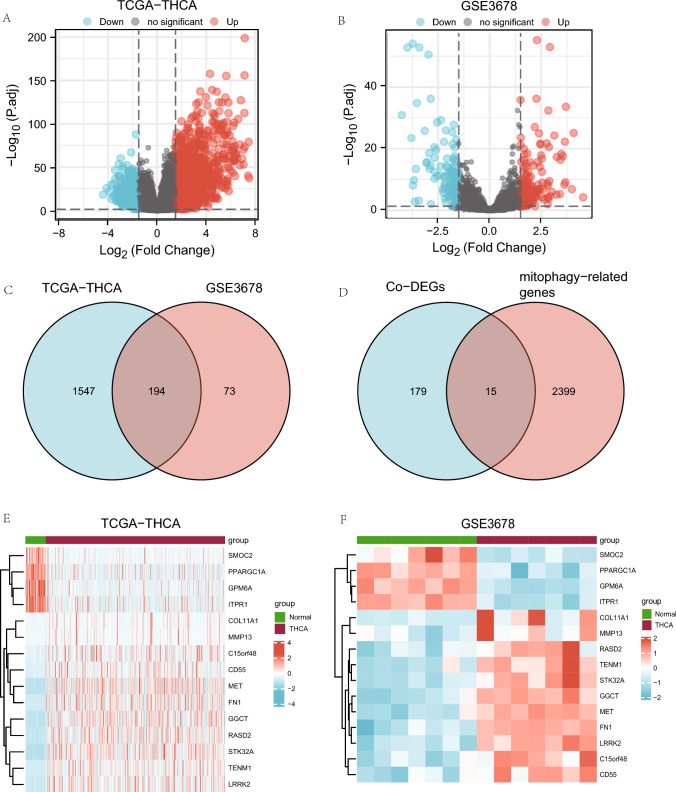
Analysis of mitophagy-associated differentially expressed genes (DEGs) in ThyC. **A** A volcano plot of DEGs from the TCGA-ThyC dataset comparing cancer tissues (grouping: tumor) and surrounding non-malignant tissues (grouping: normal). **B** A volcano plot of DEGs in ThyC from the GSE3678 dataset. **C** DEGs from both TCGA-ThyC and GSE3678 datasets were analyzed for co-DEGs using a Venn diagram. **D** A Venn diagram of shared co-DEGs between two datasets and genes related to mitophagy. Co-DEGs: Common differentially expressed genes. Complex numerical heat map of mitophagy-related genes with differential expression in the TCGA-ThyC dataset (**E**) and GSE3678 dataset (**F**). Thyroid cancer: ThyC

The TCGA -ThyC data set yielded a total of 22,195 DEGs that met the |logFC|> 1.5 and P.adj 0.05 thresholds, and from these, we identified 1,741 genes related to mitophagy. The cancer group had 1,195 individuals with high expression and 546 individuals with low expression under this cutoff. Differential analysis between these two groups is presented as a volcano plot (Fig. 2A). The GSE3678 dataset has 1,719 DEGs, of which 267 genes met the |logFC|> 1.5 and *P*.adj 0.05 criteria. Under this threshold, 139 genes were upregulated, while 128 genes were downregulated. Differential analysis of this data set resulted in a volcano plot (Fig. 2B). To identify DEGs related to mitophagy, we started by taking the intersection of all DEGs from the ThyC and GSE3678 datasets. The ThyC dataset contained 194 co-DEGs that were shown, in a Venn diagram (Fig. 2C). Co-DEGs and mitophagy-related genes were then intercrossed, and a total of 15 DEGs related to mitophagy of ThyC were found (Fig. 2D). Gene names and descriptions of these 15 mitophagy-related DEGs are shown in Tables 1 and 2.

**Table 1:** List of gene symbol of mitophagy-related differentially expressed genes

Gene symbol				
GGCT	TENM1	COL11A1	RASD2	MET
PPARGC1A	SMOC2	FN1	MMP13	GPM6A
ITPR1	C15orf48	STK32A	LRRK2	CD55

**Table 2:** List of gene symbol and description of mitophagy-related DEGs

Gene_name	Description	log2FoldChange	pvalue	padj
GGCT	Gamma-Glutamylcyclotransferase	2.222200542	1.0866E-74	2.81997E-72
TENM1	Teneurin transmembrane protein 1	4.659094416	4.6859E-115	7.4426E-112
COL11A1	Collagen type XI alpha 1 Chain	5.054702423	5.69422E-38	2.73729E-36
RASD2	RASD family member 2	3.124377538	7.09195E-85	2.78812E-82
MET	MET proto-oncogene, receptor tyrosine kinase	2.669487308	5.23805E-71	1.2163E-68
PPARGC1A	PPARG Coactivator 1 alpha	−1.784754872	1.25178E-16	1.32193E-15
SMOC2	SPARC related modular calcium binding 2	−2.270758557	2.35107E-27	5.76258E-26
FN1	Fibronectin 1	5.693155783	5.5359E-106	6.1059E-103
MMP13	Matrix metallopeptidase 13	7.501081404	8.2483E-40	4.42588E-38
GPM6A	Glycoprotein M6A	−2.622331276	5.38522E-38	2.59504E-36
ITPR1	Inositol 1,4,5-trisphosphate receptor type 1	−1.844711071	3.44221E-26	7.79246E-25
C15orf48	Chromosome 15 open reading frame 48	1.567886737	9.91503E-13	7.20791E-12
STK32A	Serine/threonine kinase 32A	2.921105242	1.4505E-112	2.1332E-109
LRRK2	Leucine rich REPEAT kinase 2	4.017957621	3.81626E-95	2.52554E-92
CD55	CD55 molecule (Cromer Blood Group)	2.523918797	1.97888E-37	9.29887E-36

To the findings of the Venn diagram, the TCGA-ThyC data set (Fig. 2E) and the GSE3678 data-set (Fig. 2F) were examined for 15 mitophagy-related DEGs using the R software.

### Gene ontology (GO) analysis of DEGs related to mitophagy

**Fig. 3:**
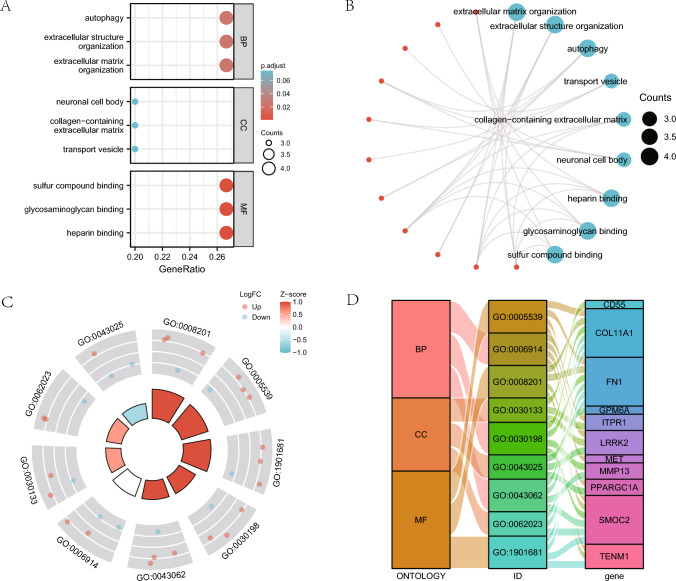
Functional gene ontology (GO) enrichment analysis of mitophagy-related DEGs. **A** The bubble plot shows findings of the GO analysis of mitophagy-related DEGs. **B** The GO analysis results of DEGs related to mitophagy are presented in the circular network diagram. **C** A circle diagram of GO analysis results combined with a logFC analysis of mitophagy-related DEGs. **D** The Sankey plot displays the findings of the GO analysis of mitophagy-associated DEGs. In the bubble plot (**A**), the color of the bubble denotes the activation or inhibition of GO terms, and the ordinates are the GO terms. Red represents activation, and blue represents inhibition. In the circular network diagram (**B**), genes are shown by red dots, whereas pathways are represented by blue circles. In the circle diagram (**C**), the red dots stand for up-regulated genes (logFC > 0) and the blue dots for down-regulated genes (logFC < 0). *GO* gene ontology, *BP* biological process, *CC* cell component, *MF* molecular function. The screening criteria for GO enrichment items were *P* < 0.05 and FDR < 0.05

We first carried out GO gene function enrichment analysis for mitophagy-related DEGs examine the relationship between 15 mitophagy-related DEGs with their BP, MF, CC and biological pathways (Table 1), and ThyC (Table 3). P and FDR values were established at 0.05 since they were deemed statistically significant. The findings demonstrated that extracellular matrix organization, autophagy, and other BPs, as well as transport vesicles, collagen-containing extracellular matrix, neuronal cell bodies, and other CCs, were enriched in Thyc. Heparin-binding,, glycosaminoglycan binding, and sulfur compound binding molecular functions were also found to be enriched in this cancer type. Bubble plots show the outcomes of the GO functional enrichment study (Fig. 3A). In addition, a ring network diagram was used to display the findings of the GO study (Fig. 3B). We next ran a combined logFC GO enrichment analysis on these 15 DEGs linking mitophagy in ThyC. Based on the enrichment analysis, the logFC value of the individual gene in the TCGA-ThyC dataset was provided for differential analysis. A Z-score corresponding to each molecule was calculated. We presented the GO enrichment analysis results of the joint logFC by circle diagram (Fig. 3C) and Sankey diagram (Fig. 3D) in the form of categories (ONTOLOGY, including BP, CC, and MF) and the relationship between the corresponding function or pathway number (ID) and the gene name.

**Table 3:** GO enrichment analysis results of mitophagy-related DEGs

Ontology	ID	Description	GeneRatio	BgRatio	pvalue	p.adjust	qvalue
BP	GO:2001028	Positive regulation of endothelial cell chemotaxis	2/15	15/18670	6.29e-05	0.025	0.016
BP	GO:0090140	Regulation of mitochondrial fission	2/15	23/18670	1.51e-04	0.025	0.016
BP	GO:1901032	Negative regulation of response to reactive oxygen species	2/15	23/18670	1.51e-04	0.025	0.016
BP	GO:1903206	Negative regulation of hydrogen peroxide-induced cell death	2/15	23/18670	1.51e-04	0.025	0.016
BP	GO:2001039	Negative regulation of cellular response to drug	2/15	23/18670	1.51e-04	0.025	0.016
CC	GO:0005604	Basement membrane	2/15	95/19717	0.002	0.074	0.044
CC	GO:0030133	Transport vesicle	3/15	392/19717	0.003	0.074	0.044
CC	GO:0062023	Collagen-containing extracellular matrix	3/15	406/19717	0.003	0.074	0.044
CC	GO:0005793	Endoplasmic reticulum-Golgi intermediate compartment	2/15	126/19717	0.004	0.074	0.044
CC	GO:0043025	Neuronal cell body	3/15	497/19717	0.006	0.074	0.044
MF	GO:0008201	Heparin binding	4/15	169/17697	1.01e-05	0.001	6.06e-04
MF	GO:0005539	Glycosaminoglycan binding	4/15	229/17697	3.33e-05	0.002	9.39e-04
MF	GO:1901681	Sulfur compound binding	4/15	250/17697	4.70e-05	0.002	9.39e-04
MF	GO:0005518	Collagen binding	2/15	67/17697	0.001	0.041	0.022
MF	GO:0005262	Calcium channel activity	2/15	123/17697	0.005	0.092	0.049

*GO* Gene Ontology, *BP* biological process, *CC* cell component, *MF* molecular function

### GSEA of the ThyC dataset

**Fig. 4:**
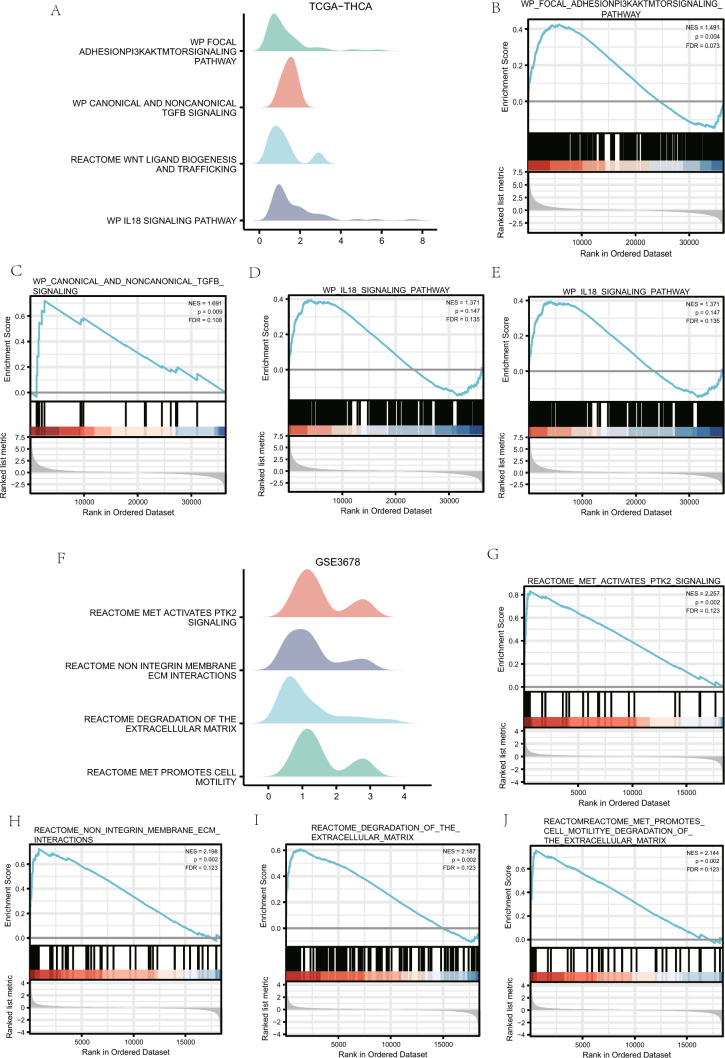
GSEA of theThyC dataset. (**A**) There were four main biological features in the GSEA of the TCGA-ThyC dataset. **B**–**E** TCGA-ThyC dataset contains significant DEGs in focal adhesion-related PI3K/Akt/mTOR signaling pathways (**B**) canonical and no-canonical TGF-β signaling (**C**), WNT ligand biogenesis and trafficking (**D**) IL-18 signaling pathway (**E**) and other pathways. (**F**) GSEA analysis of the GSE3678 dataset included four main biological characteristics. **G**–**J** DEGs were significantly enriched in MET-activated PTK2 signaling (**G**) non-integrin membrane-ECM interactions (**H**) ECM degradation (**I**) and MET-mediated cell motility (**J**) The significant enrichment screening criteria of GSEA enrichment analysis included P < 0.05 and FDR < 0.25

The GSEA was performed to investigate the connection between the expression of DEGs and BP, CC, and MF in the TCGA-ThyC and GSE3678 datasets. Both P < 0.05 and FDR < 0.25 thresholds were required for significant enrichment screening. According to these findings, genes in the focal adhesion PI3K/Akt/mTOR signaling pathway (Fig. 4B), canonical and non-canonical TGF- signaling pathways (Fig. 4C), WNT ligand biogenesis and trafficking (Fig. 4D), IL-18 signaling pathway (Fig. 4E), and extra pathways (Fig. 4A–E, Table 4) were significantly differentially expressed in the TCGA-ThyC enrichment dataset. However, DEGs in the GSE3678 dataset were significantly enriched in MET activate PTK2 signaling (Fig. 4G), and non-integrin membrane-ECM interactions (Fig. 4H). Degrading the extracellular matrix (ECM; Fig. 4I), MET promotes cell motility (Fig. 4J) and other pathways (Fig. 4F–J, Table 5).

**Table 4:** GSEA of dataset TCGA-THCA

Description	setSize	Enrichmentscore	NES	pvalue	p.adjust
REACTOME_FORMATION_OF_THE_CORNIFIED_ENVELOPE	128	0.734455925	2.378710633	0.001345895	0.069706745
REACTOME_DEGRADATION_OF_THE_EXTRACELLULAR_MATRIX	140	0.710131029	2.308891368	0.001351351	0.069706745
REACTOME_COLLAGEN_DEGRADATION	64	0.78241245	2.296585549	0.001503759	0.069706745
REACTOME_KERATINIZATION	216	0.668386606	2.28194975	0.001269036	0.069706745
REACTOME_EXTRACELLULAR_MATRIX_ORGANIZATION	300	0.636387385	2.229490948	0.001210654	0.069706745
REACTOME_ASSEMBLY_OF_COLLAGEN_FIBRILS_AND_OTHER_MULTIMERIC_STRUCTURES	61	0.753352898	2.1978989	0.00152439	0.069706745
NABA_ECM_REGULATORS	238	0.630785336	2.166338433	0.001248439	0.069706745
REACTOME_ACTIVATION_OF_MATRIX_METALLOPROTEINASES	33	0.813521776	2.15459814	0.001650165	0.069706745
PID_INTEGRIN1_PATHWAY	66	0.731640296	2.147060596	0.00149925	0.069706745
REACTOME_CELL_JUNCTION_ORGANIZATION	91	0.694749767	2.133937569	0.001436782	0.069706745
REACTOME_COLLAGEN_FORMATION	90	0.694920331	2.128994706	0.001436782	0.069706745
WP_CANONICAL_AND_NONCANONICAL_TGFB_SIGNALING	17	0.718260731	1.691020986	0.008912656	0.124689631
REACTOME_WNT_LIGAND_BIOGENESIS_AND_TRAFFICKING	26	0.637184527	1.613385402	0.017152659	0.169177882
WP_FOCAL_ADHESIONPI3KAKTMTORSIGNALING_PATHWAY	303	0.425114129	1.490649942	0.003636364	0.083720766
WP_IL18_SIGNALING_PATHWAY	273	0.394563188	1.370567279	0.014742015	0.155433977

*GSEA* gene set enrichment analysis, *TCGA* The cancer genome atlas, *THCA* thyroid cancer

**Table 5:** GSEA of dataset GSE3678

Description	setSize	enrichmentScore	NES	pvalue	p.adjust	qvalues
REACTOME_MET_ACTIVATES_PTK2_SIGNALING	30	0.831027133	2.256766192	0.002183406	0.131651557	0.123347391
REACTOME_NON_INTEGRIN_MEMBRANE_ECM_INTERACTIONS	56	0.724471893	2.197933457	0.002222222	0.131651557	0.123347391
REACTOME_DEGRADATION_OF_THE_EXTRACELLULAR_MATRIX	138	0.609086629	2.186968164	0.002283105	0.131651557	0.123347391
REACTOME_MET_PROMOTES_CELL_MOTILITY	41	0.756019945	2.144310081	0.002325581	0.131651557	0.123347391
PID_INTEGRIN1_PATHWAY	65	0.662606559	2.101523027	0.002207506	0.131651557	0.123347391
KEGG_ECM_RECEPTOR_INTERACTION	81	0.634679082	2.091044287	0.00228833	0.131651557	0.123347391
REACTOME_REGULATION_OF_INSULIN_LIKE_GROWTH_FACTOR_IGF_TRANSPORT_AND_UPTAKE_BY_INSULIN_LIKE_GROWTH_FACTOR_BINDING_PROTEINS_IGFBPS_	119	0.593328856	2.084846592	0.002325581	0.131651557	0.123347391
REACTOME_ACTIVATION_OF_MATRIX_METALLOPROTEINASES	31	0.759234987	2.075262843	0.002192982	0.131651557	0.123347391
NABA_ECM_REGULATORS	226	0.549420188	2.071691645	0.002380952	0.131651557	0.123347391
REACTOME_SYNDECAN_INTERACTIONS	25	0.782218088	2.046394931	0.002178649	0.131651557	0.123347391
REACTOME_DEFENSINS	24	0.778133951	2.021959202	0.002150538	0.131651557	0.123347391
REACTOME_ASSEMBLY_OF_COLLAGEN_FIBRILS_AND_OTHER_MULTIMERIC_STRUCTURES	61	0.64837202	2.002527836	0.002247191	0.131651557	0.123347391
REACTOME_COLLAGEN_DEGRADATION	64	0.621487987	1.963595331	0.002183406	0.131651557	0.123347391
BIOCARTA_MHC_PATHWAY	11	0.89217319	1.934279336	0.002114165	0.131651557	0.123347391
PID_SYNDECAN_1_PATHWAY	46	0.666268574	1.932009304	0.002331002	0.131651557	0.123347391

*GSEA* Gene set enrichment analysis

### The PPI network analysis

**Fig. 5:**
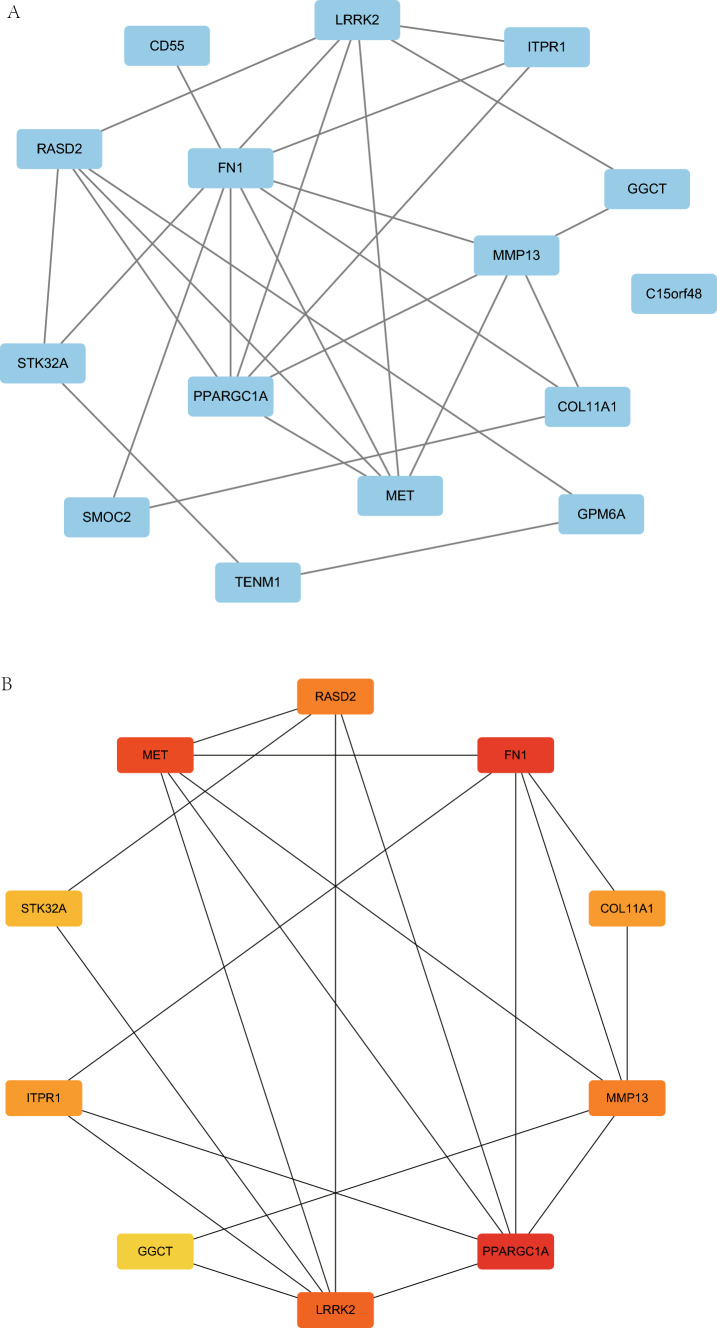
The PPI network. **A** The PPI network of mitophagy-related DEGs. **B**The PPI network of the top 10 mitophagy-related DEGs (key genes) in the MCC algorithm. The change in color of the rectangular block in the figure from yellow to red represents a gradual increase in the score. *PPI network* protein–protein interaction network, *MCC* maximal clique centrality

We examined the STRING database for the network analysis PPI of 15 mitophagy-related DEGs (see Table 1 for details). The minimal needed interaction tally was selected as the confidence parameter in the STRING database, setting a value of 0.150 for low confidence. A PPI network of 15 mitophagy-related DEGs was constructed and visualized using the Cytoscape software (Fig. 5A). We then used the MCC algorithm to determine the grade of mitophagy-related DEGs throughout the PPI network that were linked to other PPI network nodes. The top ten genes with the highest marks were then shown as the important genes (hub genes) for ThyC after we sorted the mitophagy-related DEGs by their scores (Fig. 5B). The 10 mitophagy-related DEGs include: *PPARGC1A, FN1, MET, LRRK2, MMP13, RASD2, COL11A1, ITPR1, STK32A* and *GGCT*. The specific gene score levels are shown in Table S2.

### Construction of a predictive model for DEGs associated with mitophagy and analysis of their differential expression patterns

**Fig. 6:**
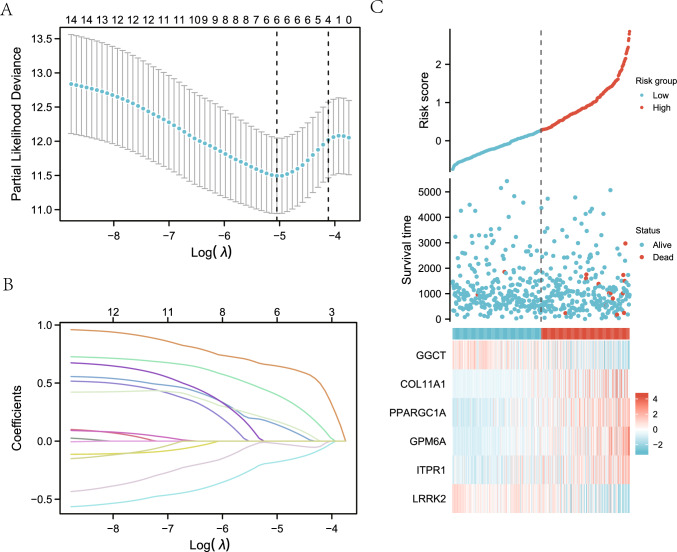
Construction of a prognostic model of DEGs related to mitophagy and analysis of their differential expressions. **A** Diagram of a LASSO regression predictive model for mitophagy-related DEGs. Variable trajectory plots from the LASSO regression predictive model (**B**) and the risk factor map (**C**). The likelihood deviation value of LASSO regression is represented by the ordinate of the LASSO regression prognosis model (**A**) and the situation after taking the lambda (λ) coefficient log of the penalty term in LASSO regression as a default is shown by the X-axis log value at the bottom of the figure. The number on the top X-axis indicates how many variables are under each having non-zero corresponding coefficients

A prediction model was created using LASSO regression analysis to assess the prognostic significance of 15 mitophagy-related DEGs (see Table 1 for details) in the TCGA-ThyC dataset (Fig. 6A). Six genes (*GGCT, COL11A1, PPARGC1A, GPM6A, ITPR1* and *LRRK2*) were identified using this model. To create the LASSO variable trajectory graphics, we also displayed the LASSO regression findings (Fig. 6B). We then visualized the grouping of samples in the constructed LASSO model using a risk factor plot (Fig. 6C). The risk score estimated by the samples' model was grouped by the median, and the survival time and survival outcome of the clinical samples in the TCGA-ThyC data set were displayed by a dot plot. Finally, the heat map was plotted to visualize the expression of the prognostic DEGs related to mitophagy in the LASSO regression prognosis model.

### Prognostic analysis of mitophagy-associated DEGs

**Fig. 7:**
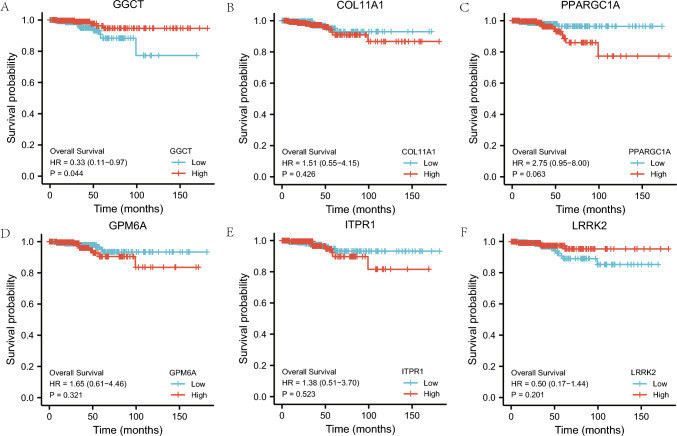
Prognostic analysis of mitophagy-related DEGs. **A**–**F** The KM curve of prognostic analysis of mitochondrial autophagy-related differentially expressed *GGCT* (**A**), *COL11A1* (**B**), *PPARGC1A* (**C**), *GPM6A* (**D**), *ITPR1* (**E**), and *LRRK2* (**F**) genes. *KM curve* Kaplan–Meier curve

For prognostic analysis, the LASSO regression model was comprised of six mitophagy-related prognostic DEGs,namely (*GGCT, COL11A1, PPARGC1A, GPM6A, ITPR1,*and *LRRK2*). The prognostic survival KM curves of these DEGs were plotted individually (Fig. 7 A–F), and they were deemed statistically significant at P < 0.05. GGCT (P = 0.044, Fig. 7A) was identified as a DEG with a significant prognostic value..

### The ROC curve analysis of prognostic DEGs related to mitophagy

**Fig. 8:**
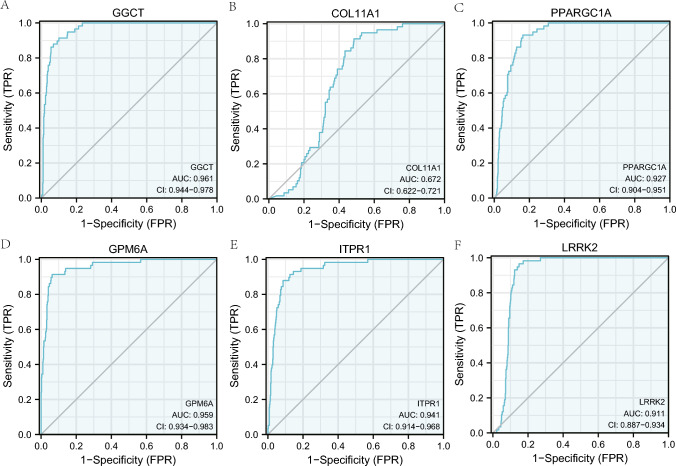
ROC curves of prognostic DEGs related to mitophagy. **A**–**F** The ROC curve results of mitophagy related prognostic DEGs, *GGCT* (**A**), *COL11A1* (**B**), *PPARGC1A* (**C**), *GPM6A* (**D**), *ITPR1* (**E**), and *LRRK2* (**F**) in the TCGA-ThyC dataset are shown. ROC: receiver operating characteristic curve

The expression variations of the six mitophagy-related prognostic DEGs (*GGCT, COL11A1, PPARGC1A, GPM6A, ITPR1,* and *LRRK2*) were further analyzed using ROC curves in the TCGA-ThyC dataset (Fig. 8A–F). According to the ROC curve analysis, the LASSO regression model was used to select those six genes. In addition to *COL11A1* (AUC = 0.672, Fig. 8B), expressions of *GGCT* (AUC = 0.961, Fig. 8A), *PPARGC1A* (AUC = 0.927, Fig. 8C), *GPM6A* (AUC = 0.959, Fig. 8D), *ITPR1* (AUC = 0.941, Fig. 8E), and *LRRK2* (AUC = 0.911, Fig. 8F) were also indicated a strong connection with ThyC pathogenesis.

### Evaluation of the prognostic potential of mitophagy-related DEGs

**Fig. 9:**
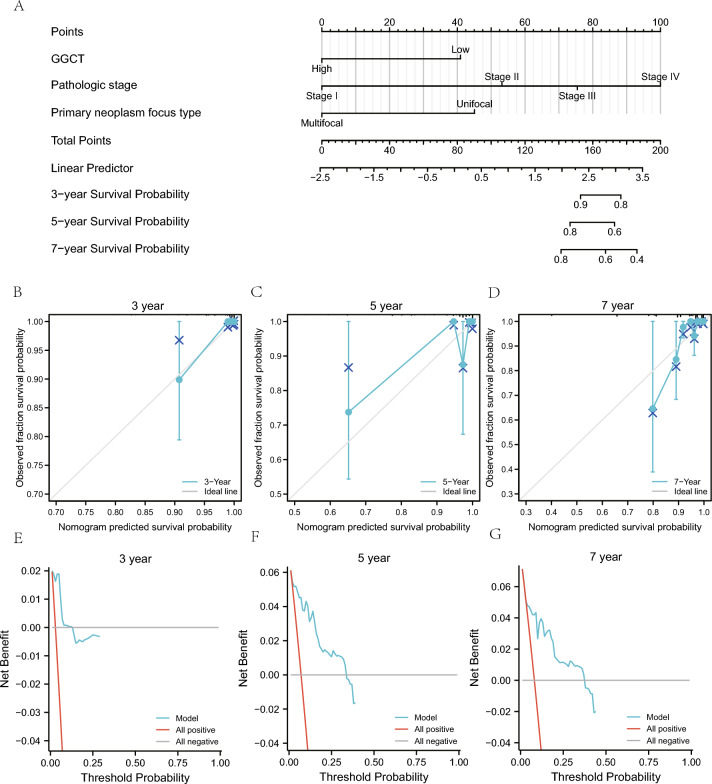
Prognostic analysis of mitophagy-related DEGs. **A** Nomogram for prognostic DEGs associated with mitophagy in univariate and multivariate Cox regression analyses. **B**–**D** The 3-year (**B**), 5-year (**C**) and 7-year (**D**) calibration plots for univariate and multivariate Cox regression analyses of th nomogram model. **E**–**G** The 3-year (**E**), 5-year (**F**) and 7-year (**G**) DCA plots of the LASSO-Cox regression prognostic model. The DCA plot's Y-axis depicts the net benefit, while the X-axis represents the probability threshold or threshold probability. *DCA* decision curve analysis

We conducted a statistical analysis of the clinical data of ThyC patients derived from the TCGA-Thy C dataset, based on the correlation with *GGCT* gene expression, to further support the LASSO regression prognostic model (Table 6). Then we analyzed the correlation between the *GGCT* expression and different clinical variables and prognosis in the TCGA-ThyC dataset. First, we performed univariate COX regression analysis on the *GGCT* gene expression and different clinical variables and selected the factors with P < 0.1 for constructing the multivariate Cox regression model. Our findings demonstrated a substantial correlation between pathological stage, residual tumor volume, initial neoplasm focal type, and prognosis (P < 0.05; Table 7). Then, we developed the nomogram and performed a nomogram analysis to assess the model's predictive power (Fig. 9A). Furthermore, we calibrated the nomogram of univariate and multivariate Cox regression models for 3-year (Fig. 9B), 5-year (Fig. 9C), and 7-year (Fig. 9D) prognoses and drew a calibration curve (Fig. 9B–D). The 3-year blue line (Fig. 9B) was the closest to the gray ideal circumstance line, suggesting that the 3-year prediction effect might be superior to that of the 5- and 7-year predictions. The clinical applicability of this model was then assessed and presented using DCA at 3-year (Fig. 9E), 5-year (Fig. 9F), and 7-year (Fig. 9E–G) survival rates. The blue line representing the model was stable and higher than the red line for all positives and the gray line for all negatives. The range of x values for 5-year (Fig. 9F) was close to that of 7-year (Fig. 9G), and the 3-year (Fig. 9E) prognosis range was the smallest, indicating that the model's ability to predict the survival rate seems to improve over time.

**Table 6:** Patient Characteristics of THCA patients in the TCGA datasets

Characteristic	Low expression of GGCT	High expression of GGCT	p
n	255	255	
T stage, n (%)			0.004
T1	82 (16.1%)	61 (12%)	
T2	92 (18.1%)	75 (14.8%)	
T3	73 (14.4%)	102 (20.1%)	
T4	7 (1.4%)	16 (3.1%)	
N stage, n (%)			< 0.001
N0	134 (29.1%)	95 (20.7%)	
N1	87 (18.9%)	144 (31.3%)	
Pathologic stage, n (%)			0.039
Stage I	149 (29.3%)	137 (27%)	
Stage II	33 (6.5%)	19 (3.7%)	
Stage III	49 (9.6%)	64 (12.6%)	
Stage IV	23 (4.5%)	34 (6.7%)	
Histological type, n (%)			< 0.001
Classical	160 (31.4%)	204 (40%)	
Follicular	84 (16.5%)	17 (3.3%)	
Other	4 (0.8%)	5 (1%)	
Tall Cell	7 (1.4%)	29 (5.7%)	
Extrathyroidal extension, n (%)			< 0.001
No	193 (39.2%)	145 (29.5%)	
Yes	49 (10%)	105 (21.3%)	
Thyroid gland disorder history, n (%)			0.003
Lymphocytic thyroiditis	43 (9.5%)	31 (6.9%)	
Nodular hyperplasia	42 (9.3%)	26 (5.8%)	
Normal	123 (27.2%)	162 (35.8%)	
Other, specify	17 (3.8%)	8 (1.8%)	

*TCGA* The cancer genome atlas, *THCA* thyroid cancer

**Table 7:** COX regression to identify clinical features associated with OS

Characteristics	Total(N)	Univariate analysis		Multivariate analysis	
		Hazard ratio (95% CI)	P value	Hazard ratio (95% CI)	P value
GGCT	510				
Low	255	Reference			
High	255	0.332 (0.114–0.969)	**0.044**	0.177 (0.021–1.536)	0.116
T stage	508				
T1	143	Reference			
T2	167	1.030 (0.171–6.191)	0.974	0.493 (0.024–10.207)	0.648
T3	175	1.602 (0.309–8.304)	0.575	0.191 (0.009–3.972)	0.285
T4	23	11.518 (2.303–57.620)	**0.003**	2.040 (0.015–278.917)	0.776
M stage	295				
M0	286	Reference			
M1	9	4.258 (0.909–19.952)	0.066	0.000 (0.000-Inf)	0.999
Pathologic stage	508				
Stage I	286	Reference			
Stage II	52	5.380 (0.753–38.446)	0.094	24.669 (0.540–1125.993)	0.100
Stage III	113	9.733 (2.018–46.944)	**0.005**	37.297 (1.999–696.040)	**0.015**
Stage IV	57	18.760 (3.601–97.751)	** < 0.001**	34.649 (0.240–5005.316)	0.162
Residual tumor	448				
R0	390	Reference			
R1	54	4.033 (1.214–13.402)	**0.023**	8.705 (1.348–56.227)	**0.023**
R2	4	0.000 (0.000-Inf)	0.998	0.000 (0.000-Inf)	0.999
Primary neoplasm focus type	500				
Multifocal	233	Reference			
Unifocal	267	3.950 (0.891–17.506)	0.071	13.980 (1.068–183.001)	**0.044**

Bold represents statistically significant values (p < 0.05)

*OS* overall survival

### GSVA of prognostic DEGs related to mitophagy

**Fig. 10:**
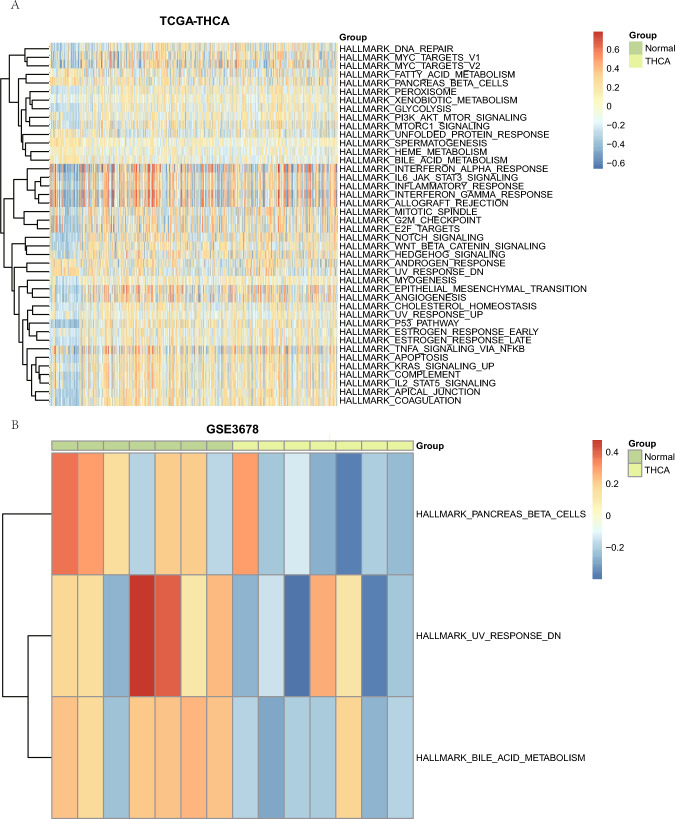
GSVA of prognostic DEGs related to mitophagy. Heatmap of functional scores in GSVA for datasets TCGA-ThyC (**A**) and GSE3678 (**B**). Samples are exhibited on the horizontal axis, and the biological function is on the vertical axis. The node color represents the activation or inhibition of the corresponding function; blue represents the inhibition, and red represents the activation. GSVA: gene set variation analysis

We then performed GSVA on the prognostic DEGs’ (*GGCT*, *COL11A1*, *PPARGC1A*, *GPM6A*, *ITPR1*, and *LRRK2*) expressions related to mitophagy in TCGA-ThyC and GSE3678 datasets to investigate changes in their expression levels between cancerous and neighboring non-cancerous tissue samples. A GSVA of these prognostic DEGs in the TCGA-ThyC dataset detected a total of 42 hallmark genes related to the p53 pathway, coagulation, apical junction, etc. that were differentially regulated in ThyC (Fig. 10A; Table 8). The GSVA results of prognosis-related DEGs in the GSE3678 dataset revealed three hallmark gene sets (UV-response DN, bile acid metabolism, and pancreatic function that were differentially expressed in this cancer type (Fig. 10B; Table 9).

**Table 8:** GSVA of dataset TCGA-THCA

ID	logFC	AveExpr	t	*P*.Value	adj.P.Val	B
HALLMARK_P53_PATHWAY	0.3768218	−0.004078401	10.83994615	4.55E-25	2.27E-23	46.01595841
HALLMARK_COAGULATION	0.351662583	0.026542729	10.169446	1.79E-22	4.47E-21	40.11138465
HALLMARK_APICAL_JUNCTION	0.344203346	0.006930155	9.824898314	3.48E-21	5.81E-20	37.17934661
HALLMARK_NOTCH_SIGNALING	0.346286083	6.75E-05	8.730404308	2.67E-17	3.34E-16	28.35876033
HALLMARK_WNT_BETA_CATENIN_SIGNALING	0.313712619	0.001714427	8.60273333	7.21E-17	7.21E-16	27.38117989
HALLMARK_ESTROGEN_RESPONSE_LATE	0.246999977	−0.005090355	7.406798172	4.56E-13	3.80E-12	18.78240801
HALLMARK_GLYCOLYSIS	0.230715127	−0.025801665	7.236615434	1.46E-12	1.04E-11	17.64418804
HALLMARK_SPERMATOGENESIS	−0.18400436	0.022540546	−6.962862507	9.02E-12	5.64E-11	15.85979406
HALLMARK_MYOGENESIS	0.208854604	0.006142073	6.797284334	2.64E-11	1.47E-10	14.80877068
HALLMARK_APOPTOSIS	0.248444169	0.002369153	6.604850516	8.97E-11	4.49E-10	13.61443837
HALLMARK_BILE_ACID_METABOLISM	−0.193755909	−0.017961356	−6.432515059	2.62E-10	1.19E-09	12.56992725
HALLMARK_ANGIOGENESIS	0.270638324	0.018325637	6.158580293	1.37E-09	5.70E-09	10.9590967
HALLMARK_EPITHELIAL_MESENCHYMAL_TRANSITION	0.27345893	−0.006903902	6.089616622	2.05E-09	7.57E-09	10.56324111
HALLMARK_UV_RESPONSE_DN	−0.229112411	−0.014036771	−6.084421437	2.12E-09	7.57E-09	10.53357921
HALLMARK_HEDGEHOG_SIGNALING	0.233524795	0.011435876	5.875581953	7.09E-09	2.36E-08	9.359738293
HALLMARK_PEROXISOME	0.190064602	−0.015328465	5.840142485	8.67E-09	2.71E-08	9.164147388
HALLMARK_COMPLEMENT	0.213997193	0.01071676	5.572759969	3.84E-08	1.13E-07	7.722535311
HALLMARK_PANCREAS_BETA_CELLS	−0.193223333	0.025798123	−5.242827534	2.21E-07	6.15E-07	6.027629177
HALLMARK_HEME_METABOLISM	−0.14661062	−0.024793887	−5.124415209	4.06E-07	1.07E-06	5.442245401
HALLMARK_DNA_REPAIR	0.19170106	−0.031549682	5.022204723	6.80E-07	1.70E-06	4.94678652
HALLMARK_IL2_STAT5_SIGNALING	0.191173546	0.004378273	4.991166388	7.94E-07	1.89E-06	4.79814081
HALLMARK_PI3K_AKT_MTOR_SIGNALING	0.155921715	−0.011818711	4.949434479	9.76E-07	2.22E-06	4.599616001
HALLMARK_IL6_JAK_STAT3_SIGNALING	0.22424914	0.011481255	4.657973659	3.96E-06	8.61E-06	3.255997571
HALLMARK_MITOTIC_SPINDLE	0.186334755	−0.007088363	4.476217056	9.14E-06	1.90E-05	2.456427412
HALLMARK_E2F_TARGETS	0.190872349	−0.018989981	4.34745323	1.63E-05	3.25E-05	1.907961071
HALLMARK_ESTROGEN_RESPONSE_EARLY	0.142950048	−0.010095235	4.248926297	2.50E-05	4.81E-05	1.49842185
HALLMARK_INTERFERON_ALPHA_RESPONSE	0.238409428	−0.01267727	4.178058187	3.39E-05	6.28E-05	1.209306136
HALLMARK_CHOLESTEROL_HOMEOSTASIS	0.1425821	−0.019616323	4.163300613	3.61E-05	6.45E-05	1.149676545
HALLMARK_KRAS_SIGNALING_UP	0.148578458	0.019799344	3.942928279	9.03E-05	0.000155649	0.282953813
HALLMARK_XENOBIOTIC_METABOLISM	0.114377093	0.002609885	3.881845563	0.000115512	0.000192521	0.050616844
HALLMARK_G2M_CHECKPOINT	0.16673867	−0.015764936	3.871545177	0.000120377	0.000194156	0.011777077
HALLMARK_MYC_TARGETS_V2	0.178860851	−0.031757686	3.845943568	0.000133318	0.00020831	−0.084334535
HALLMARK_INFLAMMATORY_RESPONSE	0.175841487	0.009673152	3.713911853	0.000223687	0.000335263	−0.5703618
HALLMARK_MTORC1_SIGNALING	0.139487603	−0.024405819	3.708987262	0.000227979	0.000335263	−0.588176985
HALLMARK_MYC_TARGETS_V1	0.146721117	−0.023428019	3.341085028	0.000887891	0.001268416	−1.855055876
HALLMARK_ALLOGRAFT_REJECTION	0.164462076	0.003513032	3.135468432	0.001802173	0.002503018	−2.507660456
HALLMARK_INTERFERON_GAMMA_RESPONSE	0.170036845	−0.006403879	3.104776621	0.001996626	0.002698144	−2.601639665
HALLMARK_FATTY_ACID_METABOLISM	−0.107174508	−0.032426209	−2.788434403	0.005469218	0.00719634	−3.51801593
HALLMARK_UV_RESPONSE_UP	0.085839529	−0.016148619	2.745502217	0.006228212	0.007984887	−3.635008025
HALLMARK_UNFOLDED_PROTEIN_RESPONSE	−0.095138408	−0.042398343	−2.639344916	0.008528822	0.010661027	−3.916691488
HALLMARK_TNFA_SIGNALING_VIA_NFKB	0.108173626	−0.002192032	2.22853702	0.026225206	0.031981959	−4.904261649
HALLMARK_ANDROGEN_RESPONSE	−0.085541964	0.001682967	−2.187375992	0.029111511	0.034656561	−4.99419144

*GSVA* Gene set variation analysis

**Table 9:** GSVA of dataset GSE3678

ID	logFC	AveExpr	t	*P*.value	adj.P.Val	B
HALLMARK_UV_RESPONSE_DN	−0.3304021	0.021909874	−2.095288209	0.036563958	0.776356381	−4.12495255
HALLMARK_BILE_ACID_METABOLISM	−0.318883011	−0.003726451	−2.064102972	0.039437281	0.776356381	−4.143971016
HALLMARK_PANCREAS_BETA_CELLS	−0.289367178	−0.01935285	−1.983860898	0.047726627	0.776356381	−4.191596063

*GSVA* Gene set variation analysis

### IHC analysis of prognostic DEGs related to mitophagy

**Fig. 11:**
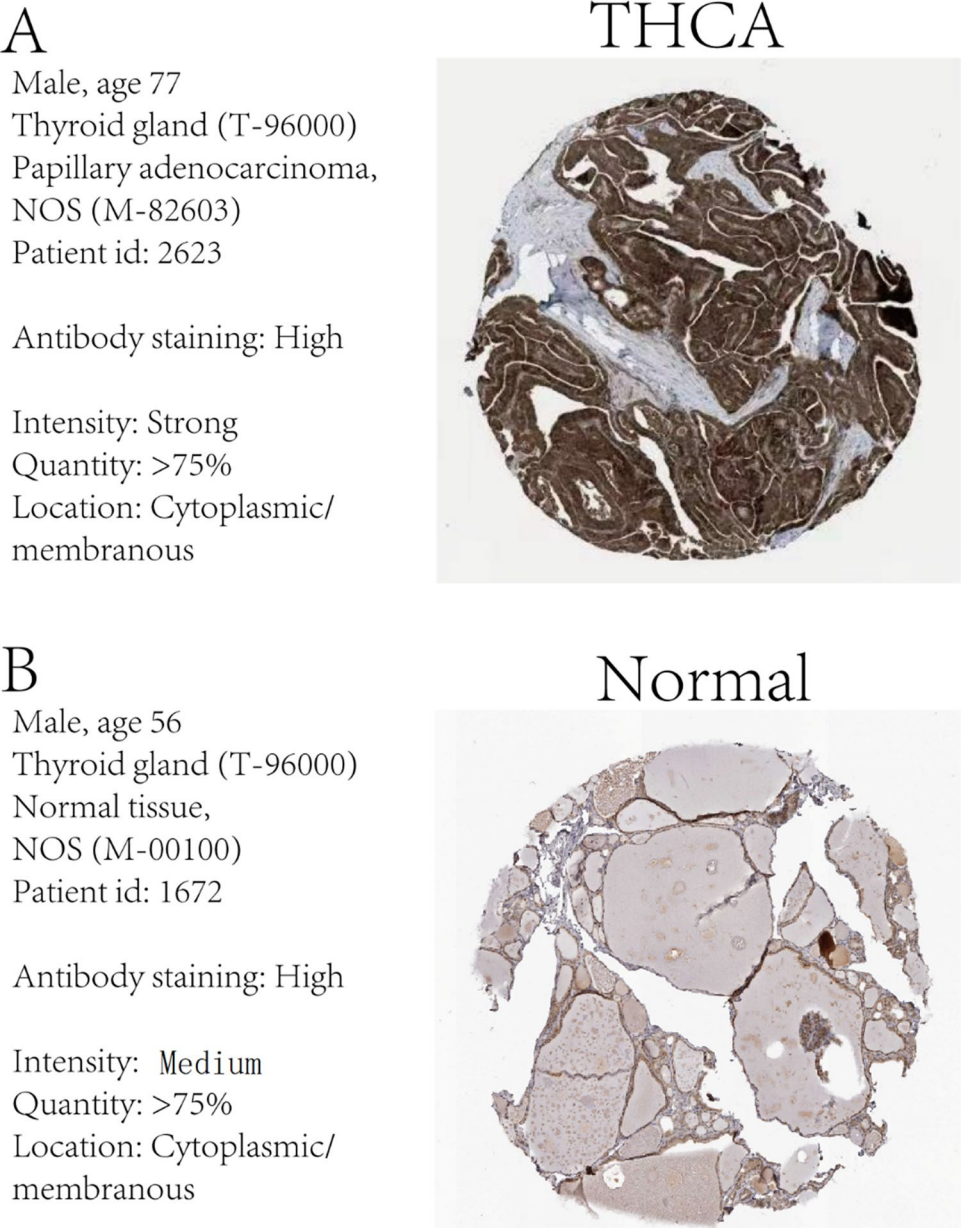
Immunohistochemical (IHC) analysis of the GGCT gene in ThyC. **A** GGCT expression in tumor tissue, **B** GGCT expression in normal tissue

The human protein atlas (HPA) database was utilized to perform an IHC analysis of the expression of the most significant prognostic DEG *GGCT* expression related to mitophagy in both normal and cancerous thyroid tissue samples. The IHC analysis consistently demonstrated that the *GGCT* gene expression was higher in the ThyC tissue than that in the normal thyroid cells in control tissues in relation to mitophagy activation (Fig. 11B). The expression level of the DEG *GGCT* was significantly higher in ThyC tissues (Fig. 11A).

## Discussion

ThyC pathology is considered the most frequently diagnosed and prevalent type of endocrine malignancy. Although the majority of ThyC patients exhibit a satisfactory prognosis, some patients have been diagnosed with metastatic ThyC type [38]. Therefore, ThyC patients must undergo a clinical assessment of metastatic risk factors and prognostic outcomes in a timely manner. Currently, the American Joint Committee on Cancer recommended TNM staging, including both clinical and pathological staging, for ThyC patients, as the most robust approach.

Autophagy can act as a double-edged sword since this pathway is involved in both tumorigenic and antitumorigenic mechanisms. While mitophagy is an autophagic process that particularly manages mitochondrial stress and degeneration. It is crucial to maintain an optimal number of mitochondria in a cell for its normal physiological energy metabolism. There has been a paucity of clinical and mechanistic investigations regarding the underlying regulatory roles of mitophagy in ThyC pathogenesis, even though this pathwayhas been linked to diagnostic and prognostic indicators in many cancer types, including lung cancer [39], breast cancer [40], and glioma [41]. Mussazhanova et al. [42] and Dabravolski et al. [43] have initially reported the pathological involvement of mitophagy in inducing ThyC pathology, however, they did not identify DEGs and their varying effects on the prognosis of ThyC patients. In this context, it’s worth mentioning that this is the first study to identify mitophagy-related potential DEGs in ThyC and their differential roles in predicting the risk factor and prognosis of ThyC patients using the two largest cancer databases—TCGA and GEO. Moreover, we revealed six mitophagy-associated genes (*GGCT*, *COL11A1*, *PPARGC1A*, *GPM6A*, *ITPR1*, and *LRRK2*) that exhibited significant differential expression levels in ThyC. Next, we constructed a nomogram model including these genetic factors to precisely predict the risk and prognosis of ThyC patients. Among these genes, the *GGCT* gene was found to be the most potential one. Hence, we validated the nomogram model based on the expression patterns of *GGCT* in ThyC versus control tissues.

Several studies have also suggested several mitophagy-associated diagnostic and prognostic biomarkers of ThyC. For instance, Han et al. [44] have discovered that ThyC growth and occurrence are highly correlated with anomalies in autophagy-related genes. They identified a set of five autophagy-related DEGs (*CX3CL1*, *CDKN2A*, *ATG9B*, *ITPR1*, and *DNAJB1*) that could be linked to the overall survival of ThyC patients after analyzing 26 DEGs from the TCGA database. Shan et al. [45] used the TCGA and HADb databases to analyze the original ThyC data and reported about 1,166 autophagy-related differentially expressed non-coding RNAs(nc-RNAs). Moreover, they found nine non-coding RNAs (AC092279.1, AC096677.1, DOCK9-DT, LINC02454, AL136366.1, AC008063.1, AC004918.3, LINC02471, and AL162231.2) that were significantly correlated with the prognosis of ThyC patients. In addition, Jia et al. [46] exploited the TCGA-ThyC dataset for the CIBERSORT algorithm analysis identifying 22 infiltrating immune cell types and screening 42 and 64 immune gene pairs (IGPs), respectively in the normal tumor group and the non-recurrence group. The number of immune cells was found to be significantly correlated with the tumor staging and relapse conditions for markers such as ASCC3MAP3K7 and ATF2-SOCS5. However, here, we identified 6 mitophagy-related DEGs that were significantly associated with ThyC pathology, and 5 of them had AUC values greater than 0.91.

In our analyses, differential expressions of *COL11A1*, *PPARGC1A*, *ITPR1*, *LRRK2*, and *GGCT* genes were found to be correlated with ThyC pathology, however, *GPM6A* did not exhibit any significant association with mitophagy in ThyC.

Type XI collagen (COL11A1) is a minor component of hyaline cartilage fibers. The *COL11A1* gene is overexpressed in many cancer types, including oral cancer and colorectal cancer. It has been implicated that the *COL11A1* gene, particularly the T allele of rs1763347 and rs2229783, could be associated with PTC [47].

The peroxisome proliferator-activated receptor gamma coactivator 1-α (PPARGC1A) is involved in energy metabolism and immunity. *PPARGC1A* has been shown to play a role in coordinating mitochondrial quality control mechanisms, including mitophagy, by affecting the expression of various genes involved in the regulation of mitophagy [48–50]. Huang et al. have reported that *PPARGC1A* level may play a critical role in the onset and progression of the ATC type of ThyC by preventing immune cell infiltration into the tumor [18]. The direct mechanistic link between *PPARGC1A* and mitophagy remains an area of ongoing research. Future studies are needed to clarify the precise mechanism by which PPARGC1A affects mitophagy.

The inositol 1,4, 5-trisphosphate receptor 1 (ITPR1), which is characterized as the direct target of hypoxia-inducible factor 2α (HIF-2α), has been reported to induce autophagy. Peng et al. have demonstrated that the long non-coding RNA SLC26A4-AS1 can promote ITPR1-mediated autophagy induction by recruiting ETS1, and thereby preventing the onset and progression of PTC [19].

The leucine-rich repeat kinase 2 (LRRK2) multi-domain protein contains a guanosine triphosphate (GTP) hydrolase domain, which is important for the function of the Ras complex. The study by Jiang revealed that *LRRK2* silencing could inhibit the JNK signaling activation, triggering cell cycle arrest and apoptosis in ThyC [51]. Therefore, the tumorigenic effects of *COL11A1* and *LRRK2* overexpression and antitumorigenic effects of *PPARGC1A* and *ITPR1* overexpression in ThyC in our study were consistent with previous studies. However, we could not reveal the crosstalk between *GPM6A* differential expression and ThyC. We plan to investigate this connection in future studies separately.

The enzyme glutamylcyclotransferase (GGCT), involved in glutathione metabolism, consists of 188 amino acids. *GGCT* overexpression has been reported to promote cancer cell growth in several cancers, including breast cancer, ovarian cancer, cervical cancer, lung cancer, bladder cancer, and colon cancer [52]. While, the downregulation of *GGCT* can inhibit the aggressive phenotype of a variety of cancers, and *GGCT* knockout cells exhibit morphological changes in cells, epithelial-mesenchymal transition (EMT), and induction of senescence, autophagy, and apoptosis, thereby inhibiting cancer cell proliferation and promoting cell death [53]. In this line *GGCT* overexpression has been closely linked to severe clinical features in ThyC, rendering poor prognosis. Mechanistically, microRNA miR-205-5p directly binds to the *GGCT* mRNA mediating its degradation and exerting an antitumor effect [17]. Zhang et al. suggest that the interaction between *GGCT* and mitochondrial protein 9 (MRPL9) regulates the MAPK/ERK pathway activation promoting the proliferation and metastatic properties of PTC cells [54]. More importantly, *GGCT* expression variation was found to have the highest diagnostic potential (AUC:0.961; 95% CI 0.944–0.978) among other mitophagy-related DEGs in our study. Therefore, we further validated the LASSO regression model and nomogram for prognostic analysis based on the *GGCT* expression. The K-M survival curve analysis also confirmed that ThyC patients with higher expression levels of *GGCT* exhibited significantly different prognostic outcomes compared to those who had relatively lower expressions of *GGCT*, in this cohort. Further, the DCA results pointed out that the precision of the prognosis prediction model increased with a longer time. The *GGCT* expression was found to associate with the pathological stage, residual tumor volume, and the primary lesion type in ThyC and significantly correlated with the prognosis of these patients. Finally, IHC analysis confirmed that ThyC patients’ tumor tissues had an overall higher level of *GGCT* expression compared to that in the non-cancerous control tissues.

In cells, mitophagy plays an essential role in maintaining mitochondrial health, as well as energy homeostasis, to support normal physiological cellular functions. Overactivation of the mitophagy pathway can be deleterious to cell health and survival because damaged mitochondria cannot balance oxidative phosphorylation, resulting in enhanced cellular oxidative stress [55] and mitochondrial membrane depolarization. Mitophagy plays a dual role in carcinogenesis. In the pathway enrichment analysis, we found that adherence to the TGFβ and PI3K/AKT/mTOR signaling pathways induced the overexpression of mitophagy-related genes to inhibit ThyC, which was in agreement with previous findings [56–58]. Therefore, we hypothesize that mitophagy might be beneficial for improving the clinical outcomes of ThyC patients.

In this study, we established a valid and reliable mitophagy-based predictive model for Thy, and this model could be exploited to assess the risk score of newly diagnosed and at-risk ThyC patients. A higher value over the baseline would indicate the risk level of that patient. Additionally, the nomogram model included the pathological staging and primary tumor type scoreing of ThyC patients, allowing the likelihood of predicting the patient's 3-, 5-, and 7-year OS rates.

However, due to the incomplete GEO clinical data, our nomogram model could not be validated multiple times in other datasets. In vitro experiments, further studies are needed to explore the upstream and downstream mechanisms of these differential genes. Also, the downstream effectors of mitophagy-related DEGs remain unknown. Therefore, a statistically large cohort of ThyC cases from multi-center and multi-platform databases is warranted for further clinical validation of this nomogram in the future.

## Conclusion

In conclusion, we identified six DEGs, namely *GGCT*, *COL11A1*, *PPARGC1A*, *GPM6A*, *ITPR1*, and *LRRK2* related to mitophagy by screening through the TCGA-ThyC dataset for the first time by bioinformatics analysis, the *GGCT* gene expression was found to be the most potent predictor in this set of genes and we successfully used the *GGCT* gene expression to validate the risk prediction precision of our nomogram model. The prognosis of ThyC patients can be accurately predicted using this model. This study thus a new perspective on how to improve the prognosis of ThyC. The outcomes of this study, in conjunction with experiments, can be used to further increase the prediction accuracy of the nomogram model in the future.

### Supplementary Information

Additional file1 (CSV 375 KB)

Additional file2 (DOCX 13 KB)

## Data Availability

Data for this study can be obtained by contacting the corresponding author upon reasonable request.
