# Supplementary material for: Development of a thyroid cancer prognostic model based on the mitophagy-associated differentially expressed genes
Source: Discov Oncol. 2023 Sep 14;14:173. doi: 10.1007/s12672-023-00772-6 (PMC10501032; doi:10.1007/s12672-023-00772-6)
Supplement: Supplementary file 2 — Additional file2 (DOCX 13 KB) [file 12672_2023_772_MOESM2_ESM.docx]

| Rank | Name | Score |
| --- | --- | --- |
| 1 | PPARGC1A | 16 |
| 2 | FN1 | 13 |
| 3 | MET | 12 |
| 4 | LRRK2 | 11 |
| 5 | MMP13 | 9 |
| 5 | RASD2 | 9 |
| 7 | COL11A1 | 4 |
| 7 | ITPR1 | 4 |
| 9 | STK32A | 3 |
| 10 | GGCT | 2 |
